# Vitamin D as a mediator in the J-shaped association between serum uric acid and all-cause and cardiovascular mortality in patients with cardiovascular–kidney–metabolic syndrome: A prospective cohort study

**DOI:** 10.1097/MD.0000000000049346

**Published:** 2026-06-26

**Authors:** Yannv Qu, Ling Wang, Zhiwei Li, Li Liu, Yansun Sun

**Affiliations:** a Geriatrics Department, Peking University Shenzhen Hospital, Shenzhen Peking University-The Hong Kong University of Science and Technology Medical Center, Shenzhen, China; b Medical School Department, Shenzhen university, Shenzhen City, China; c Geriatrics Department, Peking University Shenzhen Hospital, Shenzhen, China; d Medical Records Department, The Chinese University of Hong Kong Shenzhen Medical Centre, Shenzhen, China.

**Keywords:** all-cause mortality, cardiovascular mortality, cardiovascular-kidney-metabolic syndrome, National Health and Nutrition Examination Survey, serum uric acid, vitamin D

## Abstract

The relationship between serum uric acid (UA) and mortality in patients with cardiovascular-kidney-Metabolic (CKM) syndrome remains unclear. We hypothesized that elevated UA increases mortality nonlinearly and is partly mediated by vitamin D. Using NHANES data (2007–2016) from 25,979 adults with CKM stages 0 to 4, we performed Cox regression, threshold analysis, and causal mediation analysis. During a median follow-up of 93.0 months, 2264 all-cause and 689 cardiovascular deaths occurred. A J-shaped relationship existed between UA and mortality. Each 1 mg/dL increase in UA increased fully adjusted risks of all-cause (HR = 1.06, 95% CI: 1.02–1.09) and cardiovascular mortality (HR = 1.11, 95% CI: 1.04–1.18). Thresholds were identified at 6.6 mg/dL and 7.1 mg/dL. Participants aged ≤60 years showed higher all-cause mortality risk (HR = 1.15, 95% CI: 1.06–1.25). Serum 25(OH)D negatively correlated with UA (β = −0.064, *P* < .00001) and mediated 14.55% of its effect on all-cause mortality. High UA beyond specific thresholds predicts mortality in CKM syndrome, partially mediated by vitamin D. Interventions on UA and vitamin D may improve outcomes.

**Key Points**

- The association between serum uric acid levels and mortality is nonlinear, with an increased risk observed beyond 6.6 mg/dL.
- Higher serum uric acid is linked to elevated all-cause and cardiovascular mortality, especially in younger adults (≤60 years).
- Vitamin D mediates approximately 14.55% of the adverse effect of uric acid on mortality.

## 1. Introduction

The cardiovascular-kidney-metabolic (CKM) syndrome is a complex interplay of cardiovascular disease (CVD), chronic kidney disease (CKD), and metabolic disorders.^[1]^ This triad of conditions often coexists and exacerbates each other, leading to a significant increase in mortality risk.^[2]^ Epidemiological evidence indicates that approximately 90% of US adults fulfill the criteria for the CKM syndrome, with 9.2% falling into stage 4.^[3]^ This underscores the syndrome’s widespread prevalence and the stagnant progress in addressing it. Advanced stages of CKM syndrome are linked to heightened morbidity and mortality rates, thereby emphasizing the critical need for effective preventive measures.^[2]^ As research continues to evolve, it is becoming increasingly evident that a comprehensive understanding of the interplay between these conditions is essential for the effective management of individuals affected by CKM syndrome.

Among the various factors implicated in this syndrome, hyperuricemia has garnered considerable attention due to its association with adverse cardiovascular and renal outcomes.^[4]^ Previous studies have shown that elevated serum uric acid (UA) levels are linked to a higher risk of cardiovascular events,^[5,6]^ heart failure,^[7]^ and CKD progression,^[8]^ which in turn contribute to increased mortality.^[5,9]^ However, the exact nature of this relationship has long been a subject of debate within the scientific community. For instance, Liu et al have suggested a more complex, nonlinear association in metabolic dysfunction-associated fatty liver disease.^[10]^ Additionally, a J-shaped relationship between serum UA levels and all-cause and cardiovascular mortality has been identified in patients with diabetic kidney disease.^[11]^ Hu et al revealed a U-shaped relationship between serum UA levels and all-cause as well as cause-specific mortality in US adults.^[12]^

Vitamin D, as a hormone-like substance widely involved in calcium and phosphorus metabolism, inflammation regulation, and heart and kidney metabolic homeostasis, has attracted considerable attention for its potential roles in diabetes, kidney, and CVDs. Numerous epidemiological studies have linked low levels of 25-hydroxyvitamin D (25[OH]D) to increased all-cause and cardiovascular mortality, especially in high-risk populations such as those with obesity, abnormal glucose metabolism, and CKD. However, the evidence regarding whether vitamin D is a key mediator or modifier in hyperuricemia or diseases characterized by high UA levels (such as gout and related cardiorenal metabolic disorders) remains inconsistent, and there is a lack of large-scale randomized intervention trials targeting hyperuricemic populations to verify causality and therapeutic effects.

Therefore, this study was designed to explicitly address this gap. Beyond examining the nonlinear association between serum UA and mortality, we formally tested the hypothesis that serum 25(OH)D mediates a significant portion of this relationship, using a prespecified mediation analysis framework.

## 2. Materials and methods

### 2.1. Data source and population

The participants analyzed in this study were sourced from the National Health and Nutrition Examination Survey (NHANES) conducted between 2007 and 2016. NHANES is a national survey initiated to gather information on the health and nutritional status of the US population. The survey design details and data files can be accessed at the official NHANES website (https://www.cdc.gov/nchs/nhanes/). Utilizing a complex multistage probability sampling method, NHANES ensures that the collected data represent the entire nation. Our research strictly followed the ethical principles outlined in the Declaration of Helsinki and was endorsed by the Ethics Review Board of the National Center for Health Statistics.

**Figure 1.**
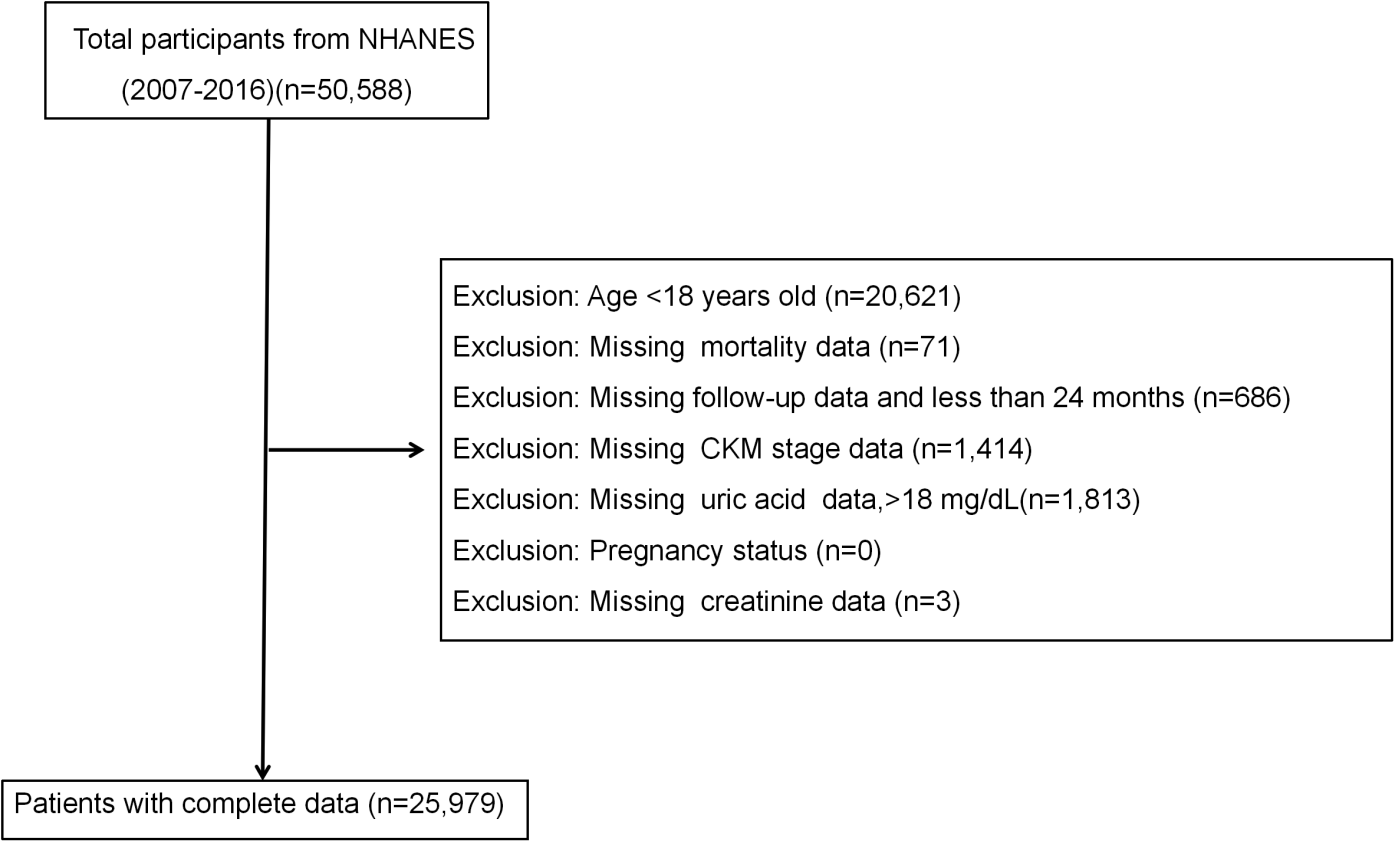
Flowchart of study population. CKM = cardiovascular–kidney–metabolic, NHANES = National Health and Nutrition Examination Survey.

In this prospective cohort study, we analyzed NHANES data from 2007 to 2016, reviewing data from five 2-year cycles and conducting a further analysis on five 2-year cycles spanning the same period. To ensure the robustness of our results, we applied the following exclusion criteria based on the study requirements and data availability: participants aged under 18 years (n = 20,621), those with missing mortality data (n = 71), individuals with incomplete follow-up data or a follow-up duration of <24 months (n = 686), participants with missing CKM stage data (n = 1414), cases with missing serum UA data or levels exceeding 18 mg/dL (n = 1813), and participants with missing creatinine data (n = 3). Importantly, no participants were excluded due to pregnancy status (Fig. 1).

### 2.2. CKM syndrome stages 0 to 4

The CKM syndrome, a systemic disorder marked by pathological interactions between the cardiovascular system, CKD, and metabolic risk factors, was classified into stages 0 to 4 based on participants’ health status. Stage 0 indicates the absence of CKM health risk factors. Stage 1 is characterized by excessive or dysfunctional obesity. Stage 2 is defined by the presence of metabolic risk factors or a moderate to high risk of CKD. Stage 3 involves subclinical CVD, which is determined by either a high predicted 10-year CVD risk or a very high-risk CKD stage. Stage 4 includes individuals with self-reported established CVD, such as coronary heart disease, angina, myocardial infarction, heart failure, or stroke. A high 10-year CVD risk is classified as 20% or greater according to the Predicting risk of cardiovascular disease events equation.^[13]^ CKD classification followed the Kidney Disease Improving Global Outcomes criteria, utilizing estimated glomerular filtration rate (eGFR) and urine albumin-creatinine ratio (UACR).^[14]^ The eGFR was calculated using the 2021 race- and ethnicity-free Chronic Kidney Disease Epidemiology Collaboration creatinine equation.^[15]^ For more details on these equations and additional staging criteria, refer to Tables S1 and S2, Supplemental Digital Content 1.

### 2.3. Outcome variables

The primary outcome was all-cause mortality, and the secondary outcome was cardiovascular mortality. Mortality data were obtained by linking records with the National Death Index. Specifically, we utilized the NHANES Public Use Linked Mortality File, current as of December 31, 2019. This file employs a probabilistic matching technique to align with the National Death Index from the National Center for Health Statistics. The cause of death was determined based on the International Statistical Classification of Diseases, 10th Revision. All-cause mortality refers to deaths due to any cause (010). Cardiovascular mortality includes deaths due to heart diseases (054–068) and cerebrovascular diseases (070).

### 2.4. Exposure variable

Serum UA levels were measured using a uricase enzymatic assay performed on a Roche/Hitachi Modular P analyzer.

### 2.5. Covariates

Demographic information, physical examinations, laboratory tests, lifestyle habits, and medical conditions were obtained from participants. Demographic variables included age, sex, race, education level, marital status, and poverty income ratio. Physical measurements encompassed BMI, waist circumference, and standing height. Laboratory data recorded were eGFR, UACR, UA, fasting blood glucose, glycated hemoglobin A1c (HbA1c), triglyceride (TG), total cholesterol, high-density lipoprotein cholesterol, and low-density lipoprotein cholesterol (LDL-C). Lifestyle factors and medical conditions included smoking status, alcohol intake, physical activity, and use of medications such as antihypertensive drugs, antihyperglycemics, insulin, and statins. Hypertension was defined as a medical diagnosis or ongoing antihypertensive treatment. Metabolic syndrome (MeTS) was diagnosed if ≥3 of the following criteria were met: waist circumference ≥ 102 cm (men) or ≥88 cm (women); high-density lipoprotein cholesterol < 40 mg/dL (men) or <50 mg/dL (women); TG ≥ 150 mg/dL; fasting glucose ≥ 100 mg/dL. Diabetes was identified by fasting blood glucose > 126 mg/dL, HbA1c ≥ 6.5%, medical diagnosis, or use of insulin/oral glucose-lowering medication. CKD classification followed Kidney Disease Improving Global Outcomes guidelines based on eGFR and UACR, categorized as low, moderate, or high risk.^[14]^ Serum 25(OH)D was measured using a standardized liquid chromatography-tandem mass spectrometry method starting with the 2007 to 08 NHANES. All measurement procedures are publicly detailed at www.cdc.gov/nchs/nhanes/ (see Table S3, Supplemental Digital Content 2).

### 2.6. Statistical methods

Continuous variables are expressed as mean ± standard deviation (SD), while categorical variables are presented as counts and percentages. To describe the study population, participants were grouped based on survival status – either surviving or deceased from all causes and cardiovascular causes. Differences between groups for continuous data were evaluated using 1-way analysis of variance (ANOVA), and categorical variables were compared with chi-square tests.

To explore the potential nonlinear relationship between serum UA levels and mortality outcomes, generalized additive models (GAMs) were employed. These models allowed for the fitting of smooth curves, revealing the existence of possible nonlinear trends and threshold effects. Missing covariate data were addressed with dummy variables.^[16]^

Hazard ratios (HRs) and 95% confidence intervals (CIs) were estimated using Cox proportional hazards regression models. Results are presented both unadjusted and adjusted for confounders selected based on clinical relevance and significant effect changes (>10%). Covariates included age; gender; race; education level; marital status; poverty income ratio; smoking; drinking; BMI; albumin; BUN; eGFR; HBA1c; FBS; LDL-C; total cholesterol; TG; UACR; total calcium; stroke; cancer; hyperlipidemia; hypertension; diabetes; CVD; antihypertensive agents; antihyperglycemic agents; CKD risk; MeTS; CKM syndrome.

### 2.7. Threshold effect analysis

A 2-piecewise linear regression approach was utilized to identify potential thresholds in the UA-mortality relationship. This involved shifting the knot across a predefined interval, selecting the point that maximized model likelihood, and testing for significance with a log-likelihood ratio test that compared the linear and piecewise models. Survival analysis was visualized with Kaplan–Meier curves, and differences between groups were tested with the log-rank test.

### 2.8. Sensitivity analyses

To ensure the robustness of findings, sensitivity analyses were performed. Missing covariate data were managed with dummy variables and appropriate imputation methods to minimize bias. Moreover, *E*-values were computed to assess the influence of possible unmeasured confounders on the observed associations. Missing variables with >5% were also addressed via multiple imputation with chained equations (5 imputations, R “mice” package), with primary analyses conducted on original data and sensitivity analyses using imputed datasets.^[17]^

### 2.9. Mediation analysis

Mediation effects of serum vitamin D on the association between UA and mortality were examined using the “mediation” package in R 4.4.3. The model adjusted for the same comprehensive set of covariates. The presence of a mediating role was established if there was a significant indirect effect, a significant total effect, and a positive proportion of the effect mediated by vitamin D. A 2-sided *P* value < .05 was deemed statistically significant. All analyses were conducted with EmpowerStats (www.empowerstats.com) and R software (version R-4.4.3, http://www.r-project.org).

## 3. Results

### 3.1. Baseline characteristics

The study population consisted of 25,979 individuals in the NHANES database (2007–2016) with CKM syndrome stages 0 to 4. During a median follow-up of 93.0 months, 2,264 participants experienced all-cause mortality, and 689 experienced cardiovascular mortality (Table 1). Notably, among those who experienced all-cause mortality, the mean age was significantly higher at 69.4 years compared to 48.5 years for those who did not (*P* < .001). A higher prevalence of male participants was observed among those who died, with 56.7% versus 48.7% (*P* < .001). Racial disparities were evident, particularly in non-Hispanic Whites, who constituted 61.0% of the deceased group compared to 41.8% in the non-deceased group (*P* < .001). Individuals with lower educational attainment and socioeconomic status were more prevalent in the mortality group. Moreover, comorbidities such as diabetes, hypertension, and CVD were significantly associated with mortality; for instance, 64.6% of those who died had hypertension (*P* < .001; Table 1). Tables S4 and S5, Supplemental Digital Content 3 in Supplementary, present participant characteristics categorized by CKM syndrome stage 0 to 4 and UA tertiles.

**Table 1:** Baseline characteristics of patients with CKM syndrome stage 0 to 4 concerning all-cause and cardiovascular mortality (N = 25,979).

		All-cause mortality			Cardiovascular mortality		
		0	1	*P* value	0	1	*P* value
N	Mean + SD	23,714	2264		25,289	689	
Age, yr	48.5 ± 17.9	46.5 ± 17.0	69.4 ± 12.5	<.001	47.9 ± 17.6	71.4 ± 11.4	<.001
Gender, n (%)				<.001			<.001
Male	12,646 (48.7%)	11,362 (47.9%)	1284 (56.7%)		12,248 (48.4%)	398 (57.8%)	
Female	13,332 (51.3%)	12,352 (52.1%)	980 (43.3%)		13,041 (51.6%)	291 (42.2%)	
Race, n (%)				<.001			<.001
Mexican American	4163 (16.0%)	3976 (16.8%)	187 (8.3%)		4116 (16.3%)	47 (6.8%)	
Other Hispanic	2848 (11.0%)	2694 (11.4%)	154 (6.8%)		2801 (11.1%)	47 (6.8%)	
Non-Hispanic White	10,846 (41.8%)	9464 (39.9%)	1382 (61.0%)		10,419 (41.2%)	427 (62.0%)	
Non-Hispanic Black	5282 (20.3%)	4850 (20.5%)	432 (19.1%)		5140 (20.3%)	142 (20.6%)	
Other race – including multi-racial	1014 (3.9%)	945 (4.0%)	69 (3.0%)		996 (3.9%)	18 (2.6%)	
Non-Hispanic Asian	1825 (7.0%)	1785 (7.5%)	40 (1.8%)		1817 (7.2%)	8 (1.2%)	
Education level, n (%)				<.001			<.001
Less than high school grade	6438 (24.8%)	5635 (23.8%)	803 (35.5%)		6184 (24.5%)	254 (36.9%)	
High school grade/GED or equivalent	5720 (22.0%)	5147 (21.7%)	573 (25.3%)		5540 (21.9%)	180 (26.1%)	
Some college or above	13,197 (50.8%)	12,316 (51.9%)	881 (38.9%)		12,943 (51.2%)	254 (36.9%)	
Marital status, n (%)				<.001			<.001
Married or living with partner	15,245 (58.7%)	14,103 (59.5%)	1142 (50.4%)		14,912 (59.0%)	333 (48.3%)	
Separated or never married	10,123 (39.0%)	9005 (38.0%)	1118 (49.4%)		9767 (38.6%)	356 (51.7%)	
Poverty income ratio, n (%)				<.001			<.001
≤1.30	8887 (34.2%)	8068 (34.0%)	819 (36.2%)		8662 (34.3%)	225 (32.7%)	
1.3–1.85	3569 (13.7%)	3172 (13.4%)	397 (17.5%)		3433 (13.6%)	136 (19.7%)	
> 1.85	11,912 (45.9%)	11,004 (46.4%)	908 (40.1%)		11,623 (46.0%)	289 (41.9%)	
Smoking, n (%)				<.001			<.001
Never	14,323 (55.1%)	13,434 (56.7%)	889 (39.3%)		14,007 (55.4%)	316 (45.9%)	
Current smoker	5212 (20.1%)	4764 (20.1%)	448 (19.8%)		5102 (20.2%)	110 (16.0%)	
Ever smoker	6041 (23.3%)	5119 (21.6%)	922 (40.7%)		5778 (22.8%)	263 (38.2%)	
Drinking, drinks/yr	40.6 ± 400.0	36.8 ± 256.8	99.4 ± 1266.9	<.001	40.0 ± 391.8	70.9 ± 705.8	.185
BMI, kg/m^2^	29.2 ± 6.8	29.2 ± 6.8	29.0 ± 6.7	.084	29.2 ± 6.8	29.3 ± 6.7	.766
Standing height, cm	167.1 ± 10.2	167.2 ± 10.1	166.0 ± 10.3	<.001	167.1 ± 10.2	165.4 ± 10.2	<.001
Waist circumference, cm	99.3 ± 16.1	98.9 ± 16.1	103.0 ± 15.9	<.001	99.1 ± 16.1	103.8 ± 15.8	<.001
TG, mg/dL	155.4 ± 133.0	155.2 ± 135.0	158.0 ± 109.6	.330	155.4 ± 133.8	155.1 ± 96.7	.940
TC, mg/dL	192.7 ± 41.5	193.1 ± 41.2	187.8 ± 44.7	<.001	192.8 ± 41.4	186.9 ± 45.4	<.001
HDL-C, mg/dL	52.6 ± 16.1	52.6 ± 15.9	52.7 ± 17.7	.865	52.7 ± 16.1	52.0 ± 16.4	.269
LDL-C, mg/dL	113.6 ± 35.4	114.3 ± 35.1	106.2 ± 37.6	<.001	113.8 ± 35.3	106.0 ± 39.0	<.001
FBS, mg/dL	109.2 ± 36.5	107.8 ± 34.5	123.8 ± 51.1	<.001	108.8 ± 36.1	123.4 ± 46.1	<.001
Albumin, g/L	42.6 ± 3.4	42.8 ± 3.3	41.1 ± 3.4	<.001	42.7 ± 3.4	41.1 ± 3.4	<.001
Alkaline phosphatase, U/L	68.7 ± 23.3	68.1 ± 22.8	75.3 ± 27.4	<.001	68.6 ± 23.1	75.2 ± 28.1	<.001
BUN, mg/dL	13.4 ± 5.7	13.0 ± 5.1	17.7 ± 9.2	<.001	13.3 ± 5.5	19.0 ± 9.3	<.001
Cr, mg/dL	0.9 ± 0.4	0.9 ± 0.4	1.1 ± 0.7	<.001	0.9 ± 0.4	1.2 ± 0.9	<.001
Uric acid, mg/dL	5.5 ± 1.4	5.4 ± 1.4	6.0 ± 1.6	<.001	5.4 ± 1.4	6.1 ± 1.7	<.001
UACR, mg/g	32.9 ± 297.8	28.1 ± 274.4	84.8 ± 480.6	<.001	31.2 ± 289.9	99.9 ± 512.5	<.001
HBA1c, %	5.8 ± 1.1	5.7 ± 1.0	6.2 ± 1.3	<.001	5.7 ± 1.1	6.2 ± 1.3	<.001
Total calcium, mg/dL	9.4 ± 0.4	9.4 ± 0.4	9.4 ± 0.4	.857	9.4 ± 0.4	9.4 ± 0.4	0.541
Serum 25(OH) vitamin D, nmol/L	63.8 ± 27.3	63.6 ± 27.0	66.4 ± 30.0	<.001	63.7 ± 27.1	67.4 ± 31.8	<.001
eGFR, ml/min/1.73m^2^	95.7 ± 22.7	97.8 ± 21.4	73.1 ± 23.7	<.001	96.4 ± 22.2	69.2 ± 22.8	<.001
Stroke, n (%)	891 (3.4%)	600 (2.5%)	291 (12.9%)	<.001	790 (3.1%)	101 (14.7%)	<.001
Cancer, n (%)	2283 (8.8%)	1759 (7.4%)	524 (23.1%)	<.001	2150 (8.5%)	133 (19.3%)	<.001
Hyperlipidemia, n (%)	8435 (32.5%)	7351 (31.0%)	1084 (47.9%)	<.001	8076 (31.9%)	359 (52.1%)	<.001
Cardiovascular disease, n (%)	2035 (7.8%)	1420 (6.0%)	615 (27.2%)	<.001	1787 (7.1%)	248 (36.0%)	<.001
Hypertension, n (%)	9070 (34.9%)	7607 (32.1%)	1463 (64.6%)	<.001	8592 (34.0%)	478 (69.4%)	<.001
Diabetes, n (%)	3210 (12.4%)	2584 (10.9%)	626 (27.7%)	<.001	2994 (11.8%)	216 (31.3%)	<.001
MetS, n (%)	10,080 (38.8%)	8915 (37.6%)	1165 (51.5%)	<.001	9697 (38.3%)	383 (55.6%)	<.001
CKD risk, n (%)				<.001			<.001
Low-risk	22,470 (86.5%)	21,041 (88.7%)	1429 (63.1%)		22,073 (87.3%)	397 (57.6%)	
Moderate to high-risk	3117 (12.0%)	2445 (10.3%)	672 (29.7%)		2875 (11.4%)	242 (35.1%)	
Very high-risk	391 (1.5%)	228 (1.0%)	163 (7.2%)		341 (1.3%)	50 (7.3%)	
Gout, n (%)	1101 (4.2%)	833 (3.5%)	268 (11.8%)	<.001	1007 (4.0%)	94 (13.6%)	<.001
Liver, n (%)	963 (3.7%)	826 (3.5%)	137 (6.1%)	<.001	930 (3.7%)	33 (4.8%)	.127
Antihypertensive agents, n (%)	7865 (30.3%)	6474 (27.3%)	1391 (61.4%)	<.001	7404 (29.3%)	461 (66.9%)	<.001
Antihyperlipidemic agents, n (%)	6090 (23.4%)	5132 (21.6%)	958 (42.3%)	<.001	5774 (22.8%)	316 (45.9%)	<.001
Antihyperglycemic agents, n (%)	2331 (9.0%)	1904 (8.0%)	427 (18.9%)	<.001	2177 (8.6%)	154 (22.4%)	<.001
CKM_Stage, n (%)				<.001			<.001
Stage 0	5112 (19.7%)	4963 (20.9%)	149 (6.6%)		5093 (20.1%)	19 (2.8%)	
Stage 1	8358 (32.2%)	8114 (34.2%)	244 (10.8%)		8301 (32.8%)	57 (8.3%)	
Stage 2	9201 (35.4%)	8753 (36.9%)	448 (19.8%)		9091 (35.9%)	110 (16.0%)	
Stage 3	1171 (4.5%)	57 (0.2%)	1114 (49.2%)		770 (3.0%)	401 (58.2%)	
Stage 4	2136 (8.2%)	1827 (7.7%)	309 (13.6%)		2034 (8.0%)	102 (14.8%)	

Frequencies are expressed as absolute numbers and percentages (%); values are means (standard deviation). Among the 25,979 patients, the amount of missing values for the covariates were 610 (2.4%) for marital status, 623 (2.4%) for education level, 1610 (6.2%) for poverty income ratio, 402 (1.5%) for smoking, 10,155 (39.1%) for drinking, 13,429 (51.7%) for FBS, and 13,811 (53.2%) for LDL-C. Numbers not totaling 100% are due to missing data. Dummy variables were used to indicate missing covariate values. To examine the robustness of the results, we conducted sensitivity analyses. Dummy variables were used to indicate missing covariate values, which was performed when continuous variables were missing more than 5% of value.

ALP = alkaline phosphatase, BMI = body mass index, BUN = blood urea nitrogen, CI = confidence interval, CKD = Chronic kidney disease, CKM = cardiovascular–kidney–metabolic, DBP = diastolic blood pressure, DM = diabetes mellitus, eGFR = estimated glomerular filtration rate, FBG = f, HbA1c = hemoglobin A1c, HDL-C = high-density lipoprotein cholesterol, HR = hazard ratio, LDL-C = low-density lipoprotein cholesterol, MeTS = metabolic syndrome, SBP = systolic blood pressure, TC = total cholesterol, TG = triglyceride, UA = uric acid, UACR = urine albumin-creatinine ratio.

### 3.2. Association between UA and mortality

**Figure 2.**
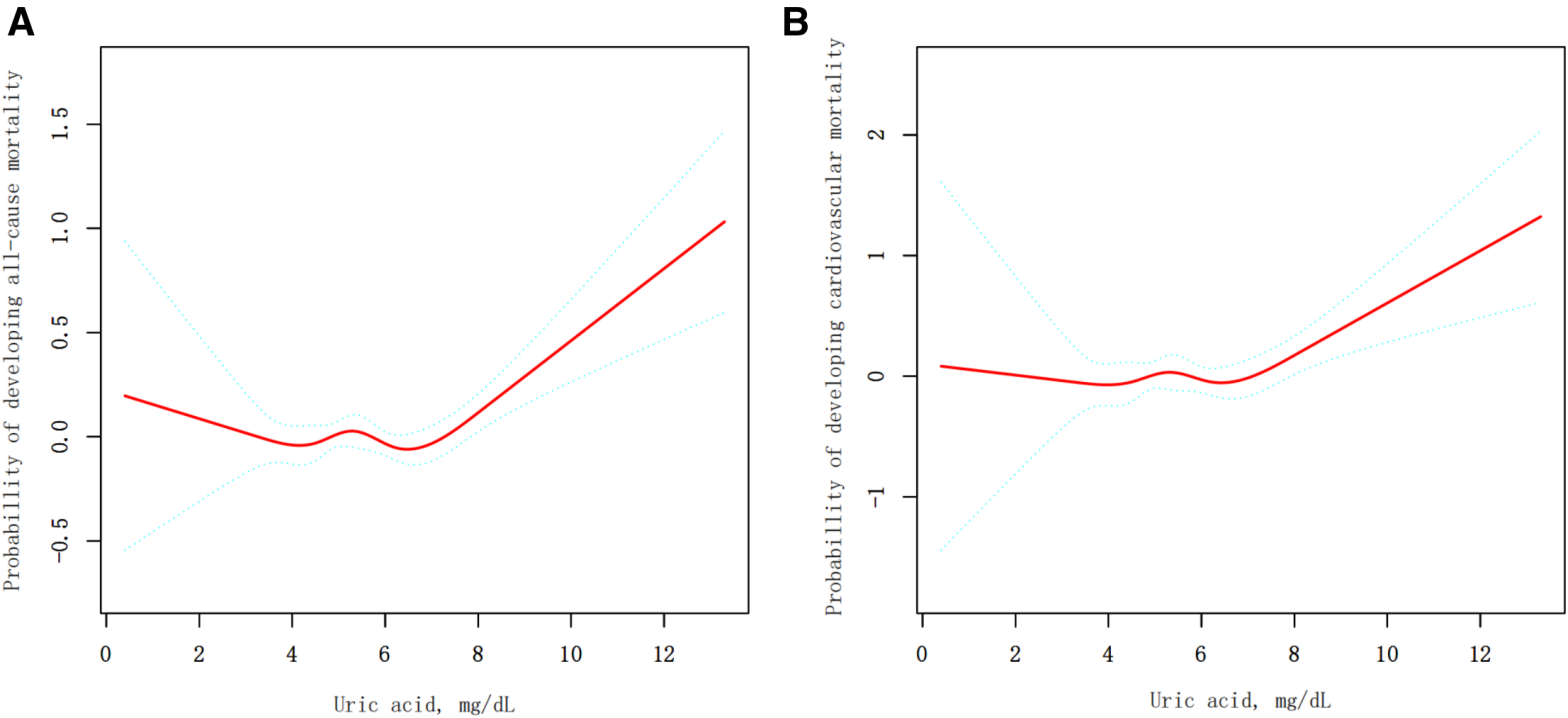
Smooth curve fitting of the association between serum UA and all-cause (A) and cardiovascular mortality (B) in a group with CKM syndrome stage 0 to 4. CKM = cardiovascular–kidney–metabolic, UA = uric acid.

A nonlinear, J-shaped relationship was observed between serum UA levels and both all-cause and cardiovascular mortality (Fig. 2). The adjusted HRs for serum UA in the fully adjusted model (adjust II) were 1.06 (95% CI: 1.02–1.09, *P* = .0020) for all-cause mortality and 1.11 (95% CI: 1.04–1.18, *P* = .0009) for cardiovascular mortality. In tertile analyses, compared to the low UA tertile, the high UA tertile was associated with increased mortality risk in the non-adjusted (HR = 2.02 [95% CI: 1.82– 2.26], *P* < .0001) and partially adjusted models (Adjust I: HR = 1.18 [95% CI: 1.05–1.32], *P* = .0040), though this association was attenuated after full adjustment (Adjust II: HR = 1.07 [95% CI: 0.94–1.23], *P* = .3184 for all-cause; HR = 1.22 [95% CI: 0.94–1.58], *P* = .1272 for cardiovascular; Table 2). The associations between serum UA indices and both all-cause and cardiovascular mortality remained unchanged compared to the primary analysis using multiple imputation (Table S6, Supplemental Digital Content 4).

**Table 2:** Cox proportional hazards regression analysis of serum UA indices concerning all-cause and cardiovascular mortality in a CKM syndrome stage 0 to 4 population.

Exposure	Non-adjusted	Adjust I	Adjust II
All-cause mortality			
UA, mg/dL	1.27 (1.24–1.30) <.0001	1.10 (1.07–1.13) <.0001	1.06 (1.02–1.09) .0020
UA tertile			
Low	Reference	Reference	Reference
Middle	1.36 (1.21–1.53) <.0001	1.00 (0.89–1.13) .9757	1.08 (0.95–1.22) .2669
High	2.02 (1.82–2.26) <.0001	1.18 (1.05–1.32) .0040	1.07 (0.94–1.23) .3184
Cardiovascular mortality			
UA, mg/dL	1.36 (1.30–1.42) <.0001	1.17 (1.12–1.24) <.0001	1.11 (1.04–1.18) .0009
UA tertile			
Low	Reference	Reference	Reference
Middle	1.42 (1.14–1.77) .0017	1.03 (0.83–1.29) .7687	1.08 (0.85–1.38) .5274
High	2.51 (2.05–3.08) <.0001	1.42 (1.15–1.75) .0011	1.22 (0.94–1.58) .1272

Data were presented as HR (95% CI), *P* value; non-adjusted model adjust for: none.

Adjust I model adjust for: age; gender; race.

Adjust II model adjust for: age; gender; race; education level; marital status; poverty income ratio; smoking; drinking; BMI; albumin; BUN; eGFR; HBA1c; FBS; LDL-C; TC; TG; UACR; total calcium; stroke; cancer; hyperlipidemia; hypertension; diabetes; cardiovascular disease; antihypertensive agents; antihyperglycemic agents; CKD Risk; MeTS; CKM syndrome.

Restrict cubic spline smoothing only applies for continuous variables.

BMI = body mass index, BUN = blood urea nitrogen, CKD = chronic kidney disease, CKM = cardiovascular–kidney–metabolic, DBP = diastolic blood pressure, DM = diabetes mellitus, eGFR = estimated glomerular filtration rate, FBG = fasting blood glucose, HbA1c = hemoglobin A1c, LDL-C = low-density lipoprotein cholesterol, MeTS = metabolic syndrome, SBP = systolic blood pressure, TC = total cholesterol, TG = triglyceride, UA = uric acid, UACR = urine albumin-creatinine ratio.

### 3.3. Threshold effect analysis

Threshold effect analysis revealed significant nonlinear associations between serum UA and both all-cause and cardiovascular mortality (Table 4). For all-cause mortality, the turning point (*K*) was 6.6 mg/dL, with HRs of 0.98 (95% CI: 0.93–1.03, *P* = .4375) for UA levels < 6.6 mg/dL and 1.15 (95% CI: 1.08–1.22, *P* < .0001) for UA levels ≥ 6.6 mg/dL. For cardiovascular mortality, the turning point (*K*) was 7.1 mg/dL, with HRs of 1.00 (95% CI: 0.92–1.10, *P* = .9465) for UA levels < 7.1 mg/dL and 1.24 (95% CI: 1.10–1.40, *P* = .0006) for UA levels ≥ 7.1 mg/dL. The likelihood ratio tests were significant for both outcomes (*P* = .001 for all-cause mortality and *P* = .021 for cardiovascular mortality; Table 3).

**Table 3:** Analysis of the threshold effect of serum UA levels on all-cause and cardiovascular mortality in patients with CKM syndrome stage 0 to 4.

UA, mg/dL outcome:	All-cause mortality	Cardiovascular mortality
Model I		
1 line effect	1.05 (1.01–1.09) .0072	1.08 (1.02–1.15) .0101
Model II		
Turning point (*K*)	6.6	7.1
<*K*	0.98 (0.93–1.03) .4375	1.00 (0.92–1.10) .9465
≥*K*	1.15 (1.08–1.22) <.0001	1.24 (1.10–1.40) .0006
*P* value for LRT test	.001	.021
95% CI for turning point	6.2–7.1	6.6–7.8
UA Z-score outcome	All-cause mortality	Cardiovascular mortality
Model I		
1 line effect	1.07 (1.02–1.13) .0072	1.12 (1.03–1.23) .0101
Model II		
Turning point (*K*)	0.8	1.15
<*K*	0.97 (0.90–1.05) .4341	1.00 (0.88–1.14) .9500
≥*K*	1.22 (1.11–1.34) <.0001	1.36 (1.14–1.61) .0006
*P* value for LRT test	.001	.021
95% CI for turning point	0.52–1.15	0.8–1.64

Data were presented as HR (95% CI), *P* value; model I, linear analysis; Model II, nonlinear analysis. Adjust for: age; gender; race; education level; marital status; poverty income ratio; smoking; drinking; BMI; albumin; BUN; eGFR; HBA1c%; FBS; LDL-C; TC; TG; UACR; total calcium; stroke; cancer; hyperlipidemia; hypertension; diabetes; cardiovascular disease; antihypertensive agents; antihyperglycemic agents; CKD Risk; MeTS; CKM syndrome.

*P* < .001 indicates that model II is significantly different from model I.

BMI = body mass index, BUN = blood urea nitrogen, CKD = chronic kidney disease, CKM = cardiovascular–kidney–metabolic, CI = confidence interval, DBP = diastolic blood pressure, DM = diabetes mellitus, eGFR = estimated glomerular filtration rate, FBG = fasting blood glucose, HbA1c = hemoglobin A1c, HR = hazard ratio, LDL-C = low-density lipoprotein cholesterol, LRT = logarithm likelihood ratio test, MeTS = metabolic syndrome, SBP = systolic blood pressure, TC = total cholesterol, TG = triglyceride, UA = uric acid, UACR = urine albumin-creatinine ratio.

**Table 4:** Linear regression of serum 25(OH) vitamin D *Z*-score and tertiles with UA levels.

Exposure	Non-adjusted	Adjust I	Adjust II
Serum 25(OH) vitamin D, nmol/L *Z*-score	−0.047 (−0.064 to −0.029) <.00001	−0.066 (−0.083 to −0.048) <.00001	−0.064 (−0.081 to −0.048) <.00001
serum 25(OH) vitamin D, nmol/L tertile			
Low	Reference	Reference	Reference
Middle	0.006 (−0.038 to 0.049) .79730	−0.072 (−0.112 to −0.032) .00040	−0.043 (−0.080 to −0.006) .02220
High	−0.067 (−0.110 to −0.024) .00232	−0.146 (−0.189 to −0.104) <.00001	−0.129 (−0.169 to −0.089) <.00001

Data were presented as HR (95% CI), *P* value; model I, linear analysis; model II, nonlinear analysis. Adjust for: age; gender; race; education level; marital status; poverty income ratio; smoking; drinking; BMI; albumin; BUN; eGFR; HBA1c%; FBS; LDL-C; TC; TG; UACR; total calcium; stroke; cancer; hyperlipidemia; hypertension; diabetes; cardiovascular disease; antihypertensive agents; antihyperglycemic agents; CKD Risk; MeTS; CKM syndrome. Spline smoothing only applies for continuous variables.

BMI = body mass index, BUN = blood urea nitrogen, CKD = chronic kidney disease, CKM = cardiovascular–kidney–metabolic, Cl = confidence interval, DBP = diastolic blood pressure, DM = diabetes mellitus, eGFR = estimated glomerular filtration rate, FBG = fasting blood glucose, HbA1c = hemoglobin A1c, HR = hazard ratio, LDL-C = low-density lipoprotein cholesterol, LRT = logarithm likelihood ratio test, MeTS = metabolic syndrome, SBP = systolic blood pressure, TC = total cholesterol, TG = triglyceride, UA = uric acid, UACR = urine albumin-creatinine ratio.

**Figure 3.**
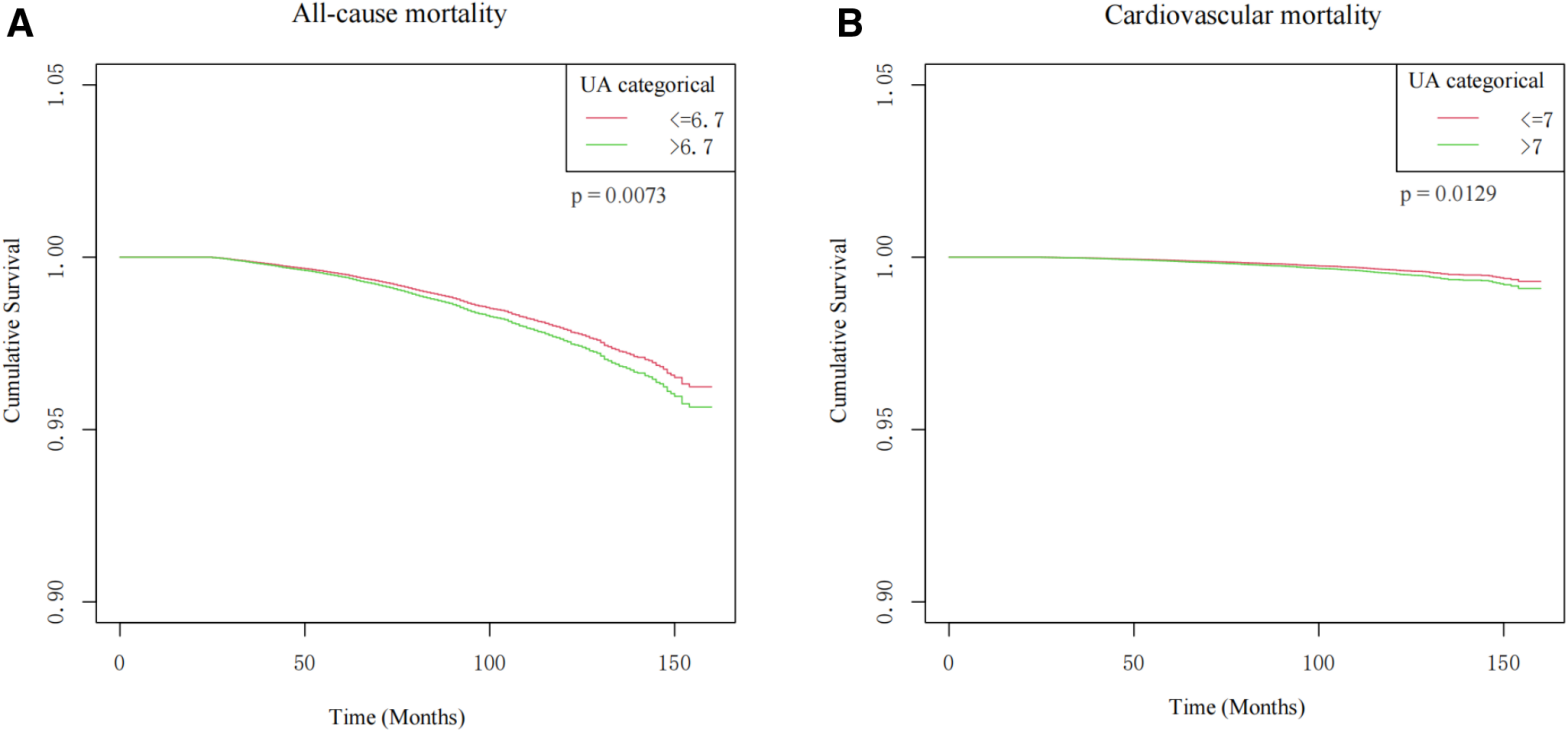
Kaplan–Meier survival curves of the association between serum UA and all-cause (A) and cardiovascular mortality (B) in a group with CKM syndrome stage 0 to 4. CKM = cardiovascular–kidney–metabolic, UA = uric acid.

The Kaplan–Meier survival curves and Cox regression analysis indicate that higher levels of UA are associated with an increased mortality risk. For all-cause mortality, individuals with UA > 6.7 have a 16% higher risk (HR = 1.1601, *P* = .0073) than those with UA ≤ 6.7. In the case of cardiovascular mortality, individuals with UA > 7 have a 29.15% higher risk (HR = 1.2915, *P* = .0129) compared to those with UA ≤ 7 (Fig. 3).

### 3.4. Subgroup analysis

**Figure 4.**
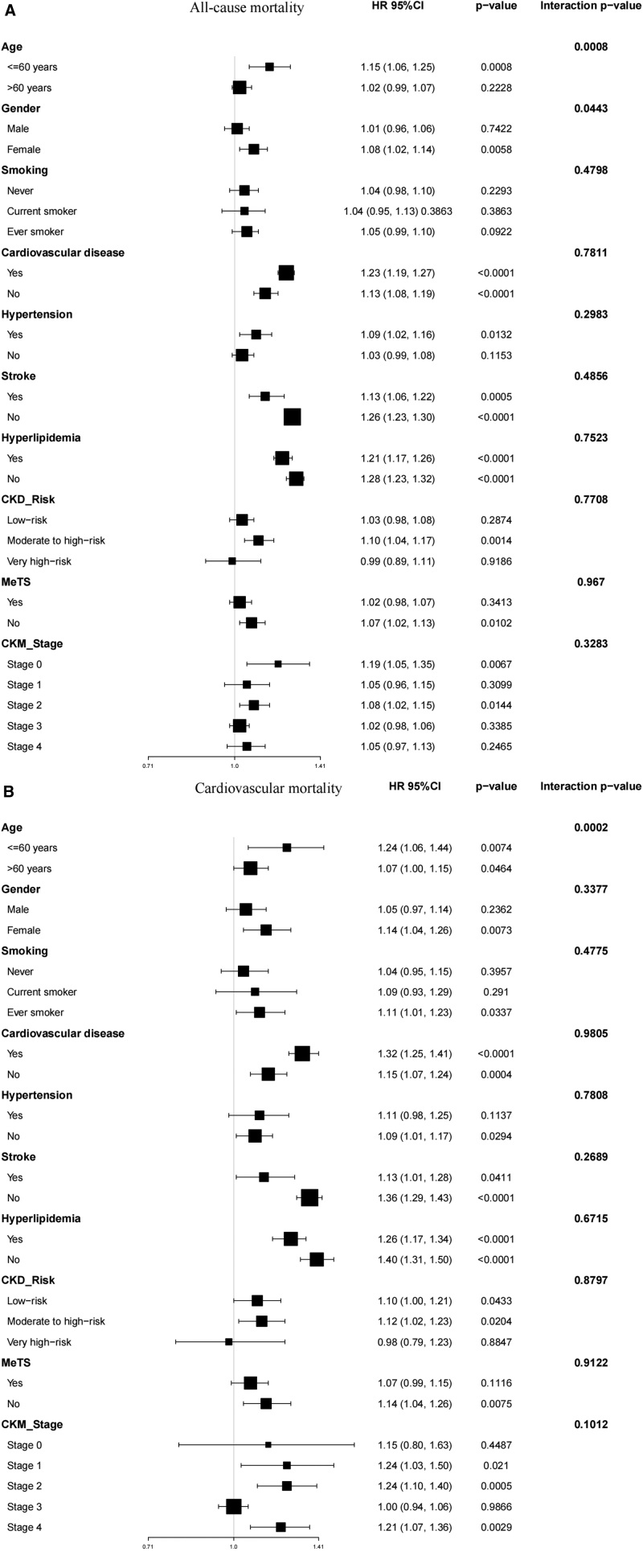
Subgroup analysis of serum UA on all-cause (A) and cardiovascular mortality (B) in a group with CKM syndrome stage 0 to 4. CI = confidence interval, CKD = chronic kidney disease, CKM = cardiovascular–kidney–metabolic, MeTS = metabolic syndrome, UA = uric acid.

The stratified analysis results show that serum UA levels are significantly associated with all-cause and cardiovascular mortality across different groups. For all-cause mortality, age demonstrates a significant interaction (*P* = .0008), with participants ≤60 years exhibiting a higher HR (1.15, 95% CI: 1.06–1.25, *P* = .0008) compared to those >60 years (HR = 1.02, 95% CI: 0.99–1.07, *P* = .2228). Similarly, for cardiovascular mortality, age reveals a significant interaction (*P* = .0002), with the ≤60 years group having a higher HR (1.24, 95% CI: 1.06–1.44, *P* = .0074) than the >60 years group (HR = 1.07, 95% CI: 1.00–1.15, *P* = .0464). Other variables such as gender, smoking status, hypertension, and MeTS show notable HRs, but their interaction *P*-values are not significant. The interaction *P*-values for CKM stages are nonsignificant for both all-cause (*P* = .3283) and cardiovascular mortality (*P* = .1012; Fig. 4A,B).

### 3.5. Associations of serum 25(OH) vitamin D with UA and mortality

Table 4 evaluated associations between serum 25(OH) vitamin D levels and UA concentrations using multiple regression models. Adjusted analyses demonstrated a significant inverse association between serum 25(OH) vitamin D *Z*-scores and UA (β = −0.064, 95% CI: −0.081 to −0.048, *P* < .00001). Tertile analysis revealed a dose-dependent pattern: the highest tertile showed the strongest reduction in UA (β = −0.129, 95% CI: −0.169 to −0.089, *P* < .00001 vs lowest tertile; Table 4).

Higher vitamin D *Z*-scores were associated with reduced all-cause mortality risk in fully adjusted models (HR = 0.91, 95% CI: 0.87–0.96, *P* = .0002). The highest tertile showed the strongest protection (HR = 0.76, 95% CI: 0.67–0.85, *P* < .0001). For cardiovascular mortality, the *Z*-score association attenuated after full adjustment (HR = 0.94, *P* = .133), while the highest tertile remained significant (HR = 0.78, 95% CI: 0.63–0.96, *P* = .022). Analyses incorporated 28 covariates, with spline adjustments for nonlinear confounders (Table 5).

**Table 5:** Cox regression of serum 25(OH) vitamin D *Z*-score and tertiles with all-cause and cardiovascular mortality.

Exposure	Non-adjusted	Adjust I	Adjust II
All-cause mortality			
Serum 25(OH) vitamin D, nmol/L *Z*-score	1.13 (1.08–1.17) <.0001	0.82 (0.79–0.86) <.0001	0.91 (0.87–0.96) .0002
Serum 25(OH) vitamin D, nmol/L tertile			
Low	Reference	Reference	Reference
Middle	0.93 (0.84–1.04) .2139	0.68 (0.61–0.76) <.0001	0.79 (0.70–0.89) .0002
High	1.24 (1.12–1.38) <.0001	0.58 (0.52–0.65) <.0001	0.76 (0.67–0.85) <.0001
Cardiovascular mortality			
Serum 25(OH) vitamin D, nmol/L *Z*-score	1.17 (1.09–1.25) <.0001	0.85 (0.78–0.92) <.0001	0.94 (0.86–1.02) .1332
Serum 25(OH) vitamin D, nmol/L tertile			
Low	Reference	Reference	Reference
Middle	0.90 (0.73–1.09) .2804	0.66 (0.54–0.81) <.0001	0.74 (0.59–0.93) .0082
High	1.36 (1.14–1.63) .0009	0.62 (0.51–0.75) <.0001	0.78 (0.63–0.96) .0217

Data were presented as HR (95% CI), *P* value; Non-adjusted model adjust for: none.

Adjust I model adjust for: age, years; gender; race.

Adjust II model adjust for: age; gender; race; education level; marital status; poverty income ratio; smoking; drinking; BMI; albumin; BUN; eGFR; HBA1c%; FBS; LDL-C; TC; TG; UACR; total calcium; stroke; cancer; hyperlipidemia; hypertension; diabetes; cardiovascular disease; antihypertensive agents; antihyperglycemic agents; CKD risk; MeTS; CKM syndrome. Restricted cubic spline were applied.

BMI = body mass index, BUN = blood urea nitrogen, CKD = chronic kidney disease, CKM = cardiovascular–kidney–metabolic, CI = confidence interval, DBP = diastolic blood pressure, DM = diabetes mellitus, eGFR = estimated glomerular filtration rate, FBG = fasting blood glucose, HbA1c = hemoglobin A1c, HR = hazard ratio, LDL-C = low-density lipoprotein cholesterol, LRT = logarithm likelihood ratio test, MeTS = metabolic syndrome, SBP = systolic blood pressure, TC = total cholesterol, TG = triglyceride, UA = uric acid, UACR = urine albumin-creatinine ratio.

### 3.6. Mediation analysis

**Figure 5.**
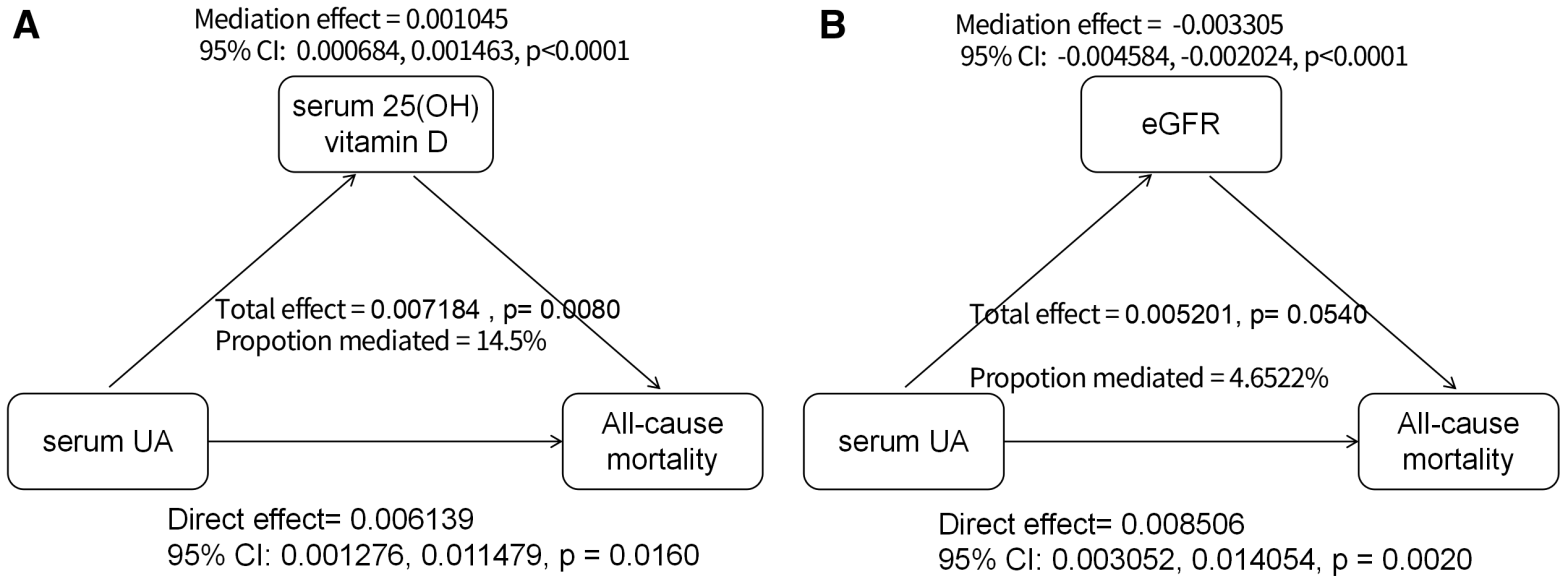
Exploratory analysis of potential pathway variables mediated by serum 25(OH) vitamin D (A) and eGFR (B) in the association between serum UA and all-cause mortality. CI = confidence interval, eGFR = estimated glomerular filtration rate, UA = uric acid.

Causal mediation analysis was conducted to evaluate the role of various biomarkers (serum 25(OH) Vitamin D and eGFR) in mediating the association between serum UA levels and all-cause mortality. For serum 25(OH) vitamin D, results showed a total effect of 0.007184 (95% CI: 0.002174–0.012558, *P* = .0080), a mediation effect of 0.001045 (95% CI: 0.000684–0.001463, *P* < .0001), and a direct effect of 0.006139 (95% CI: 0.001276–0.011479, *P* = .0160), with the proportion mediated being 14.55% (95% CI: 7.2875–42.9582, *P* = .0080; Fig. 5A). For eGFR, the total effect was 0.005201 (95% CI: −0.000026 to 0.010140, *P* = .0540), the mediation effect was −0.003305 (95% CI: −0.004584 to −0.002024, *P* < .0001), and the direct effect was 0.008506 (95% CI: 0.003052–0.014054, *P* = .0020), with the proportion mediated being −63.55% (95% CI: −3.717684 to 1.354606, *P* = .0540; Fig. 5B).

## 4. Discussion

Our study investigated the relationship between serum UA levels and mortality in a cohort of 23,635 participants from the NHANES database (2007–2016) with CKM syndrome stages 0 to 4. We identified threshold effects at 6.6 mg/dL for all-cause mortality and 7.1 mg/dL for cardiovascular mortality. Notably, in participants aged ≤60 years and those in the early stages of cardiometabolic disease (CKM), stronger inverse associations were observed. To our knowledge, this is the first study to explore the link between serum UA levels and mortality across the full spectrum of CKM syndrome stages 0 to 4. Our findings resonate with prior studies in several key aspects but also offer novel insights.

Previous studies have established that metabolic indicators such as glucose and lipid metabolism are significantly associated with mortality in CKM syndrome patients. For instance, the triglyceride-glucose correlation index,^[18]^ systemic immune-inflammation index,^[19]^ and glucose disposal rate^[20]^ have all been identified as important factors. Numerous observational studies have identified elevated serum UA as an independent risk factor for CVD,^[5,21]^ and diabetes.^[9]^

Our study contributes to this body of evidence by showing that serum UA levels are not only significantly associated with increased all-cause and cardiovascular mortality but also exhibit a clear nonlinear relationship characterized by threshold effects. This suggests that metabolic factors, including serum UA, play a crucial role in determining the mortality risk among CKM syndrome patients. This suggests that metabolic factors may play a crucial role in determining the mortality risk of CKM syndrome patients.

Moreover, in a prospective cohort study based on NHANES data among diabetic individuals, those in the highest SUA quintile had a 28% higher risk of all-cause mortality and a 41% higher risk of cardiovascular mortality compared to the lowest SUA quintile, after adjusting for multiple confounders.^[22]^ These findings are consistent with pooled analyses from the China Health and Retirement Longitudinal Study and NHANES datasets, which revealed significant positive correlations between serum UA, hyperuricemia, gout, and cardiovascular-renal-metabolic conditions, especially among participants with multiple comorbidities.^[23]^ What’s more, the nonlinear dose-response relationship identified in the restricted cubic spline analysis further elucidates the complex pathophysiology: the impact of serum UA on mortality intensifies at higher thresholds among patients with established cardiovascular-renal-metabolic conditions. Hu et al focused on US adults and found a U-shaped relationship between SUA and mortality, with an inflection point at 5.7 mg/dL.^[12]^ In contrast, our study analyzed adults with CKM stages 0 to 4 and observed a J-shaped relationship between UA and mortality, with threshold effects identified at 6.6 and 7.1 mg/dL. The nationwide retrospective cohort study from China indicates that initiating urate-lowering therapy in patients with asymptomatic hyperuricemia and type 2 Diabetes is associated with a significant reduction in all-cause mortality, as well as cardiovascular and non-CV mortality. The research shows that maintaining posttreatment serum UA levels within 360–420 μmol/L could potentially enhance this reduced mortality, suggesting a relationship between SUA levels and mortality risk.^[24]^ Importantly, our subgroup analyses highlighted that younger individuals (≤60 years) with elevated UA levels experience a more pronounced risk, suggesting that early intervention could mitigate long-term adverse outcomes.

The mechanisms underlying hyperuricemia’s role in CVD pathogenesis are multifaceted. Hyperuricemia exhibits both pathogenic and predictive roles in hypertension progression,^[25]^ and induces insulin resistance.^[26]^ Elevated serum UA levels may exacerbate cardiovascular risk by inducing lipid peroxidation and accelerating LDL-C oxidation. Hyperuricemia has been shown to impair endothelial function, thereby accelerating the early stages of atherosclerosis and preceding subsequent plaque formation.^[27]^

An interesting finding of our study is that serum 25(OH)D mediates approximately 14.55% of the effect of UA on all-cause mortality (*P* < .0001). A substantial body of epidemiological evidence links vitamin D deficiency to increased risks of all-cause mortality and CVD events and mortality.^[28]^ Since CKM syndrome involves a complex interplay among CVD, CKD, and metabolic disorders such as obesity and diabetes, understanding the role of vitamin D within this context is particularly important. Prior research has explored the relationship between vitamin D levels and mortality in populations with prediabetes, diabetes, and CKD. For example, Al-Khalidi et al found that low serum 25(OH)D concentrations (<20 ng/mL) were associated with a significant increase in both all-cause and cardiovascular-metabolic mortality among metabolically healthy obese individuals.^[29]^

Epidemiological studies further support the connection between vitamin D status and hyperuricemia-related mortality. Cohort studies focusing on patients with gout and hyperuricemia reveal an inverse relationship between serum 25(OH)D levels and all-cause mortality, with higher vitamin D levels linked to approximately a 10% reduction in cancer mortality. Additionally, vitamin D insufficiency exhibits a U-shaped association with cardiovascular mortality among hyperuricemia patients, implying that both deficiency and excess may influence risk, and that maintaining adequate vitamin D levels could help mitigate adverse outcomes.^[28]^ Cross-sectional studies using NHANES data from 2007 to 2014 have indicated that each 1 ng/mL increase in serum 25(OH)D is associated with a 2% to 5% decrease in the risk of hyperuricemia.^[30,31]^ Discrepancies across studies may be attributed to population heterogeneity and threshold effects. Variations in factors such as race, sex, and the presence of comorbidities can lead to inconsistent findings. For instance, research within Chinese populations suggests that hyperuricemia risk significantly decreases when serum 25(OH)D exceeds 28.82 ng/mL but increases below that threshold.^[32]^

While these observational patterns are consistent with a possible mediating role for vitamin D, several alternative explanations merit emphasis. First, reverse causation is plausible: more advanced CKM burden, systemic inflammation, reduced outdoor activity, malnutrition, or declining renal function could simultaneously lower 25(OH)D and raise UA, producing spurious associations with mortality. Second, UA and 25(OH)D may primarily act as biomarkers reflecting overall disease severity or frailty rather than as direct causal drivers. Third, methodological heterogeneity (differences in 25[OH]D assays and cut‑points, single baseline measurements, incomplete capture of medication use such as vitamin D supplements or urate‑lowering drugs, and unmeasured lifestyle confounders) limits causal interpretation.

This mediation indicates that vitamin D deficiency may amplify the detrimental impact of hyperuricemia and suggests potential avenues for therapeutic intervention. The mechanisms underlying these associations are multifaceted. Vitamin D plays a role in regulating UA metabolism by enhancing its renal excretion. It suppresses PTH secretion and upregulates the ABCG2 transporter, thereby promoting UA excretion.^[33]^ Conversely, hyperuricemia can inhibit renal 1α-hydroxylase, reducing active vitamin D synthesis and creating a vicious cycle of low vitamin D, high PTH, and decreased UA excretion.^[34]^ Clinical interventions using urate-lowering therapies like allopurinol have been shown to improve vitamin D status.^[35]^ Vitamin D also exerts anti-inflammatory and antioxidant effects. By inhibiting the NLRP3 inflammasome and NF-κB pathway, vitamin D reduces inflammation triggered by hyperuricemia.^[34,36]^ Its antioxidant properties help counteract the oxidative stress associated with elevated UA levels.^[36,37]^ However, mechanistic plausibility does not substitute for causal proof.

Importantly, studies indicate that maintaining adequate vitamin D levels might mitigate hyperuricemia-related mortality.^[28,36]^ For instance, in gout and hyperuricemia populations, higher serum 25(OH)D levels are associated with a lower mortality risk. Variations across populations, such as differing threshold levels for vitamin D sufficiency, may partly explain inconsistencies in prior findings.^[28]^ Nimitphong et al indicate the potential uric-lowering effect of vitamin D supplementation in patients with prediabetes and hyperuricemia,^[38]^ indicating that vitamin D status may explain a modest portion of the observed relationship. Given the observational design, single baseline biomarker measurements, potential residual confounding, and sensitivity of threshold detection to model specification, these mediation estimates should be regarded as hypothesis-generating.

## 5. Limitations and future directions

While our study benefits from a large, representative national sample and advanced analytical techniques, several important limitations must be acknowledged. First, the observational design precludes definitive causal inferences regarding the relationships between UA, vitamin D, CKM stages, and mortality. Despite extensive adjustment for covariates, residual and unmeasured confounding remains likely, particularly from lifestyle factors, dietary patterns, physical activity, and medication changes (e.g., urate-lowering or vitamin D supplementation therapies) that were either not captured in detail or were assessed only at baseline. A key limitation is the reliance on single baseline measurements of serum UA, vitamin D, eGFR, and other biomarkers. These measurements do not account for temporal variations, disease progression, or treatment effects over the follow-up period, which may introduce bias into risk estimates and limit our ability to assess dynamic relationships. Although we employed sophisticated nonlinear and mediation analyses, these results should be interpreted as identifying associations and plausible mediating pathways rather than proving causation. Finally, while the NHANES cohort is nationally representative, the generalizability of our findings to non-US populations or to healthcare settings with different management practices may be limited. Future studies incorporating repeated longitudinal measurements, more detailed time-varying confounder data, and external validation in diverse populations are needed. Ultimately, randomized controlled trials are necessary to determine whether interventions to correct vitamin D deficiency and manage UA levels can effectively improve survival in patients with CKM syndrome.

## 6. Conclusion

Elevated UA beyond certain thresholds is independently linked to increased mortality risk in CKM syndrome, partially mediated by vitamin D deficiency. Interventions addressing UA and vitamin D levels may improve prognosis in this vulnerable population.

## Acknowledgments

We thank the participants of the NHANES study and the staff involved in data collection. During the course of preparing this work, the author(s) used Kimi, Metaso, Sider, Deepl for the purpose of correct spelling, rationalize the logic of the language and modification R code. Following the use of this tool/service, the author(s) formally reviewed the content for its accuracy and edited it as necessary. The author(s) take full responsibility for all the content of this publication.

## Author contributions

**Conceptualization:** Yannv Qu, Yansun Sun.

**Data curation:** Zhiwei Li.

**Formal analysis:** Yannv Qu, Ling Wang, Zhiwei Li.

**Funding acquisition:** Yannv Qu.

**Methodology:** Yannv Qu, Ling Wang.

**Software:** Yannv Qu.

**Supervision:** Li Liu, Yansun Sun.

**Writing – original draft:** Yannv Qu, Ling Wang, Li Liu.

**Writing – review & editing:** Yansun Sun.












