# Supplementary material for: Vitamin D as a mediator in the J-shaped association between serum uric acid and all-cause and cardiovascular mortality in patients with cardiovascular–kidney–metabolic syndrome: A prospective cohort study
Source: Medicine (Baltimore). 2026 Jun 26;105(26):e49346. doi: 10.1097/MD.0000000000049346 (PMC13313750; doi:10.1097/MD.0000000000049346)
Supplement: Supplementary file 1 [file medi-105-e49346-s001.docx]

Supplementary

**Table S1 The basic PREVENT 10-year risk estimation model equations**

| **10-year CVD risk assessment equation** | |
| --- | --- |
| **Men** | **log-Odds** = -3.031168 + 0.7688528 × (age – 55) /10 + 0.0736174 × ((TC – HDL-C) × 0.02586 – 3.5) – 0.0954431 × (HDL-C × 0.02586 – 1.3) /0.3 – 0.4347345 × (min(SBP, 110) – 110) /20 + 0.3362658 × (max(SBP, 110) – 130) /20 + 0.7692857 × (if diabetes) + 0.4386871 × (if current smoker) + 0.5378979 × (min(eGFR, 60) – 60) / -15 + 0.0164827 × (max(eGFR, 60) – 90) / -15 + 0.288879 × (if using anti hypertensive medication) – 0.1337349 × (if using statin) – 0.0475924 × (if using anti-hypertensive medication) × (max(SBP, 110) – 130) /20 + 0.150273 × (if using statin) × ((TC – HDL-C) × 0.02586 – 3.5) – 0.0517874 × (age – 55) /10 × ((TC – HDL-C) × 0.02586 – 3.5) + 0.0191169 × (age – 55) /10 × (HDL-C × 0.02586 – 1.3) /0.3 – 0.1049477 × (age – 55) /10 × (max(SBP, 110) – 130) /20 – 0.2251948 × (age – 55) /10 × (if diabetes) – 0.0895067 × (age – 55) /10 × (if current smoker) – 0.1543702 × (age – 55) /10 × (min(eGFR, 60) – 60) / -15 |
|  | **Risk**= 1 / (1 + exp(-log-Odds)) |
| **Women** | log-Odds = -3.307728 + 0.7939329 × (age – 55) /10 + 0.0305239 × ((TC – HDL-C) × 0.02586 – 3.5) – 0.1606857 × (HDL-C × 0.02586 – 1.3) /0.3 – 0.2394003 × (min(SBP, 110) – 110) /20 + 0.360078 × (max(SBP, 110) – 130) /20 + 0.8667604 × (if diabetes) + 0.5360739 × (if current smoker) + 0.6045917 × (min(eGFR, 60) – 60) / -15 + 0.0433769 × (max(eGFR, 60) – 90) / -15 + 0.3151672 × (if using anti hypertensive medication) – 0.1477655 × (if using statin) – 0.0663612 × (if using anti-hypertensive medication) × (max(SBP, 110) – 130) /20 + 0.1197879 × (if using statin) × ((TC – HDL-C) × 0.02586 – 3.5) – 0.0819715 × (age – 55) /10 × ((TC – HDL-C) × 0.02586 – 3.5) + 0.0306769 × (age – 55) /10 × (HDL-C × 0.02586 × 0.02586 – 1.3) /0.3 – 0.0946348 × (age – 55) /10 × (max(SBP, 110) – 130) /20 – 0.27057 × (age – 55) /10 × (if diabetes) – 0.078715 × (age – 55) /10 × (if current smoker) – 0.1637806 × (age – 55) /10 × (min(eGFR, 60) – 60) / -15 |
|  | **Risk**= 1 / (1 + exp(-log-Odds)) |

Abbreviations: TC, total cholesterol; HDL-C, high-density lipoprotein cholesterol; SBP, systolic blood pressure; eGFR, estimated glomerular filtration rate.
