# Supplementary material for: Vitamin D as a mediator in the J-shaped association between serum uric acid and all-cause and cardiovascular mortality in patients with cardiovascular–kidney–metabolic syndrome: A prospective cohort study
Source: Medicine (Baltimore). 2026 Jun 26;105(26):e49346. doi: 10.1097/MD.0000000000049346 (PMC13313750; doi:10.1097/MD.0000000000049346)
Supplement: Supplementary file 2 [file medi-105-e49346-s002.docx]

**Supplementary**

**Table S3 **Variables, Measurement Methods, and Equipment/Techniques****

| **Variable** | **Measurement Method** | **Equipment/Technique** |
| --- | --- | --- |
| **Body Mass Index (BMI)** | Calculated from measured height and weight: BMI = weight (kg) / height² (m²) | Calibrated scales, stadiometers |
| **Systolic/Diastolic Blood Pressure (SBP/DBP)** | Mercury sphygmomanometer in seated position, with three measurements averaged for accuracy. | Mercury sphygmomanometer |
| **Serum Creatinine** | Enzymatic method | Roche/Hitachi Modular P analyzer |
| **Uric Acid** | Uricase enzymatic assay | Roche/Hitachi Modular P analyzer |
| **High-Density Lipoprotein Cholesterol (HDL-C)** | Direct enzymatic colorimetric method | Roche/Hitachi Modular P analyzer |
| **Total Cholesterol** | Enzymatic method | Roche/Hitachi Modular P analyzer |
| **Glycohemoglobin** | High-Performance Liquid Chromatography (HPLC) | Tosoh Automated Glycohemoglobin Analyzer |
| **Albumin-Creatinine Ratio (ACR)** | Urine assay | Immunoassay for albumin, enzymatic assay for creatinine |
