# Supplementary material for: Vitamin D as a mediator in the J-shaped association between serum uric acid and all-cause and cardiovascular mortality in patients with cardiovascular–kidney–metabolic syndrome: A prospective cohort study
Source: Medicine (Baltimore). 2026 Jun 26;105(26):e49346. doi: 10.1097/MD.0000000000049346 (PMC13313750; doi:10.1097/MD.0000000000049346)
Supplement: Supplementary file 3 [file medi-105-e49346-s003.docx]

Supplementary

Table S4. The baseline characteristics stratified by CKM syndrome stage 0–4.

| CKM Stage | Stage 0 | Stage 1 | Stage 2 | Stage 3 | Stage 4 | p-value |
| --- | --- | --- | --- | --- | --- | --- |
| N | 5112 | 8358 | 9201 | 1171 | 2136 |  |
| Age, years | 39.6 ± 16.4 | 42.9 ± 15.6 | 51.6 ± 15.9 | 76.5 ± 7.1 | 63.0 ± 12.8 | <0.001 |
| **Gender**, n (%) |  |  |  |  |  | <0.001 |
| Male | 2629 (51.4%) | 3959 (47.4%) | 4195 (45.6%) | 678 (57.9%) | 1185 (55.5%) |  |
| Female | 2483 (48.6%) | 4399 (52.6%) | 5006 (54.4%) | 493 (42.1%) | 951 (44.5%) |  |
| **Race**, n (%) |  |  |  |  |  | <0.001 |
| Mexican American | 588 (11.5%) | 1505 (18.0%) | 1750 (19.0%) | 88 (7.5%) | 232 (10.9%) |  |
| Other Hispanic | 548 (10.7%) | 979 (11.7%) | 1049 (11.4%) | 58 (5.0%) | 214 (10.0%) |  |
| Non-Hispanic White | 2213 (43.3%) | 3123 (37.4%) | 3682 (40.0%) | 786 (67.1%) | 1042 (48.8%) |  |
| Non-Hispanic Black | 922 (18.0%) | 1959 (23.4%) | 1728 (18.8%) | 184 (15.7%) | 489 (22.9%) |  |
| Other Race - Including Multi-Racial | 284 (5.6%) | 306 (3.7%) | 308 (3.3%) | 35 (3.0%) | 81 (3.8%) |  |
| Non-Hispanic Asian | 557 (10.9%) | 486 (5.8%) | 684 (7.4%) | 20 (1.7%) | 78 (3.7%) |  |
| **Education Level**, n (%) |  |  |  |  |  | <0.001 |
| Less Than High School Grad | 938 (18.3%) | 1850 (22.1%) | 2512 (27.3%) | 439 (37.5%) | 638 (33.3%) |  |
| High School Grad/GED or Equivalent | 994 (19.4%) | 1747 (20.9%) | 2154 (23.4%) | 294 (25.1%) | 531 (24.9%) |  |
| Some College or above | 2884 (56.4%) | 4549 (54.4%) | 4426 (48.1%) | 436 (37.2%) | 902 (42.2%) |  |
| **Marital Status** , n (%) |  |  |  |  |  | <0.001 |
| Married or Living with partner | 2709 (53.0%) | 5028 (60.2%) | 5697 (61.9%) | 583 (49.8%) | 1228 (57.5%) |  |
| Separated or Never married | 2110 (41.3%) | 3118 (37.3%) | 3399 (36.9%) | 588 (50.2%) | 908 (42.5%) |  |
| **Poverty income ratio**, n (%) |  |  |  |  |  | <0.001 |
| ≤1.30 | 1680 (32.9%) | 2704 (32.4%) | 3285 (35.7%) | 369 (31.5%) | 849 (39.7%) |  |
| 1.3~1.85 | 636 (12.4%) | 1111 (13.3%) | 1249 (13.6%) | 231 (19.7%) | 342 (16.0%) |  |
| > 1.85 | 2450 (47.9%) | 4042 (48.4%) | 4109 (44.7%) | 497 (42.4%) | 814 (38.1%) |  |
| Smoking, n (%) |  |  |  |  |  | <0.001 |
| Never | 2865 (56.0%) | 5024 (60.1%) | 5087 (55.3%) | 500 (42.7%) | 847 (39.7%) |  |
| Current smoker | 1248 (24.4%) | 1511 (18.1%) | 1804 (19.6%) | 129 (11.0%) | 520 (24.3%) |  |
| Ever smoker | 805 (15.7%) | 1683 (20.1%) | 2243 (24.4%) | 542 (46.3%) | 768 (36.0%) |  |
| Drinking, g/year | 35.9 ± 224.0 | 35.4 ± 222.1 | 47.8 ± 594.0 | 26.5 ± 156.6 | 47.8 ± 363.5 | 0.366 |
| Albumin, g/L | 44.1 ± 3.2 | 42.6 ± 3.4 | 42.2 ± 3.2 | 40.9 ± 3.4 | 41.7 ± 3.2 | <0.001 |
| Creatinine, mg/dL | 0.8 ± 0.2 | 0.8 ± 0.2 | 0.9 ± 0.4 | 1.4 ± 1.1 | 1.0 ± 0.6 | <0.001 |
| FBS, mg/dL | 96.6 ± 18.5 | 98.6 ± 20.1 | 120.5 ± 45.3 | 121.1 ± 36.6 | 124.0 ± 49.6 | <0.001 |
| HBA1c, % | 5.3 ± 0.6 | 5.5 ± 0.6 | 6.1 ± 1.3 | 6.2 ± 1.1 | 6.3 ± 1.4 | <0.001 |
| TC, mg/dL | 184.6 ± 38.4 | 193.8 ± 38.5 | 200.0 ± 43.4 | 182.0 ± 42.9 | 182.1 ± 44.4 | <0.001 |
| LDL-C, mg/dL | 107.1 ± 32.9 | 117.0 ± 33.3 | 118.4 ± 36.7 | 101.5 ± 35.0 | 102.4 ± 37.3 | <0.001 |
| HDL-C, mg/dL | 60.3 ± 16.5 | 55.7 ± 14.6 | 46.3 ± 14.4 | 51.9 ± 15.9 | 50.1 ± 16.7 | <0.001 |
| TG, mg/dL | 102.7 ± 68.0 | 120.9 ± 85.8 | 213.4 ± 173.3 | 154.2 ± 100.0 | 167.6 ± 120.0 | <0.001 |
| ALP, U/L | 62.3 ± 19.8 | 66.9 ± 22.7 | 72.3 ± 23.7 | 74.5 ± 27.1 | 72.9 ± 25.4 | <0.001 |
| Total calcium, mg/dL | 9.5 ± 0.3 | 9.4 ± 0.3 | 9.4 ± 0.4 | 9.4 ± 0.4 | 9.4 ± 0.4 | <0.001 |
| UA, mg/dL | 4.9 ± 1.2 | 5.3 ± 1.3 | 5.7 ± 1.4 | 6.2 ± 1.7 | 5.9 ± 1.5 | <0.001 |
| BUN, mg/dL | 12.1 ± 3.9 | 12.3 ± 4.1 | 13.6 ± 5.4 | 22.0 ± 10.6 | 15.8 ± 7.3 | <0.001 |
| UACR, mg/g | 6.2 ± 13.2 | 5.8 ± 5.3 | 46.6 ± 312.1 | 172.9 ± 968.1 | 71.7 ± 389.6 | <0.001 |
| serum 25(OH)D levels, nmol/L | 66.7 ± 28.0 | 61.4 ± 26.3 | 62.8 ± 26.4 | 72.0 ± 30.5 | 66.4 ± 29.1 | <0.001 |
| eGFR, ml/min/1.73m2 | 104.8 ± 17.6 | 101.7 ± 18.1 | 93.5 ± 22.1 | 59.1 ± 21.7 | 79.3 ± 22.5 | <0.001 |
| Standing height, cm | 168.2 ± 10.0 | 167.3 ± 9.9 | 166.6 ± 10.5 | 164.7 ± 10.2 | 166.8 ± 10.0 | <0.001 |
| Waist circumference, cm | 81.3 ± 6.5 | 99.6 ± 12.1 | 107.0 ± 15.3 | 102.0 ± 14.7 | 106.3 ± 16.4 | <0.001 |
| BMI | 22.2 ± 1.7 | 30.0 ± 5.5 | 32.1 ± 6.9 | 28.2 ± 5.9 | 31.0 ± 7.4 | <0.001 |
| Stroke, n (%) | 0 (0.0%) | 0 (0.0%) | 0 (0.0%) | 181 (15.5%) | 710 (33.2%) | <0.001 |
| Cancer, n (%) | 282 (5.5%) | 435 (5.2%) | 863 (9.4%) | 319 (27.2%) | 384 (18.0%) | <0.001 |
| Gout, n (%) | 54 (1.1%) | 128 (1.5%) | 474 (5.2%) | 179 (15.3%) | 266 (12.5%) | <0.001 |
| Antihypertensive agents,n (%) | 401 (7.8%) | 1125 (13.5%) | 4017 (43.7%) | 861 (73.5%) | 1461 (68.4%) | <0.001 |
| Antihyperlipidemic agents,n (%) | 378 (7.4%) | 1052 (12.6%) | 2853 (31.0%) | 521 (44.5%) | 1286 (60.2%) | <0.001 |
| Hyperlipidemia,n (%) | 792 (15.5%) | 1955 (23.4%) | 3805 (41.4%) | 561 (47.9%) | 1322 (61.9%) | <0.001 |
| Antihyperglycemic agents,n (%) | 64 (64.0%) | 152 (50.3%) | 1355 (76.6%) | 247 (67.1%) | 513 (76.5%) | <0.001 |
| DM, n (%) | 100 (2.0%) | 302 (3.6%) | 1769 (19.2%) | 368 (31.4%) | 671 (31.4%) | <0.001 |
| Hypertension, n (%) | 582 (11.4%) | 1425 (17.0%) | 4641 (50.4%) | 882 (75.3%) | 1540 (72.1%) | <0.001 |
| Cardiovascular disease, n (%) | 0 (0.0%) | 0 (0.0%) | 0 (0.0%) | 370 (31.6%) | 1665 (77.9%) | <0.001 |
| Liver, n (%) | 104 (2.0%) | 204 (2.4%) | 453 (4.9%) | 47 (4.0%) | 155 (7.3%) | <0.001 |
| CKD Risk, n (%) |  |  |  |  |  | <0.001 |
| Low-risk | 51 12 (100.0%) | 8358 (100.0%) | 6959 (75.6%) | 537 (45.9%) | 1504 (70.4%) |  |
| Moderate to high-risk | 0 (0.0%) | 0 (0.0%) | 2156 (23.4%) | 432 (36.9%) | 529 (24.8%) |  |
| Very high-risk | 0 (0.0%) | 0 (0.0%) | 86 (0.9%) | 202 (17.3%) | 103 (4.8%) |  |
| MeTS, n (%) | 0 (0.0%) | 0 (0.0%) | 8215 (89.3%) | 607 (51.8%) | 1258 (58.9%) | <0.001 |
| All-cause mortality, n (%) | 149 (2.9%) | 244 (2.9%) | 448 (4.9%) | 1114 (95.1%) | 309 (14.5%) | <0.001 |
| Cardiovascular mortality, n (%) | 19 (0.4%) | 57 (0.7%) | 110 (1.2%) | 401 (34.2%) | 102 (4.8%) | <0.001 |

Abbreviations: HR, hazard ratio; CI,confidence interval; BMI, body mass index; SBP, systolic blood pressure; DBP, diastolic blood pressure; DM, diabetes mellitus; eGFR, estimated glomerular filtration rate; FBG, fasting blood glucose; HbA1c, hemoglobin A1c; HDL-C, high-density lipoprotein cholesterol; LDL-C, low-density lipoprotein cholesterol; TC, total cholesterol; TG, triglyceride; UACR, urinary albumin creatinine ratio；ALP, alkaline phosphotase; UA, uric acid; BUN, blood urea nitrogen; CKD , Chronic kidney disease；MeTS, metabolic syndrome; CKM, Cardiovascular-Kidney-Metabolic Syndrome.

Frequencies are expressed as absolute numbers and percentages (%); values are means (standard deviation). Among the 25,979 patients, the amount of missing values for the covariates were 610 (2.4%) for marital status, 623 (2.4%) for education level, 1,610 (6.2%) for poverty income ratio, 402 (1.5%) for smoking, 10,155 (39.1%) for drinking, 13,429 (51.7%) for FBS, and 13,811 (53.2%) for LDL-C. Numbers not totalling 100% are due to missing data. Dummy variables were used to indicate missing covariate values.To examine the robustness of the results, we conducted sensitivity analyses. Dummy variables were used to indicate missing covariate values, which was performed when continuous variables were missing more than 5% of value.
