# Supplementary material for: Vitamin D as a mediator in the J-shaped association between serum uric acid and all-cause and cardiovascular mortality in patients with cardiovascular–kidney–metabolic syndrome: A prospective cohort study
Source: Medicine (Baltimore). 2026 Jun 26;105(26):e49346. doi: 10.1097/MD.0000000000049346 (PMC13313750; doi:10.1097/MD.0000000000049346)
Supplement: Supplementary file 4 [file medi-105-e49346-s004.docx]

Table S6 Cox proportional hazards regression analysis of serum uric acid indices concerning all-cause and cardiovascular mortality in a CKM syndrome stage 0–4 population(Using Multiple Imputation).

| Exposure | Non-adjusted | Adjust I | Adjust II |
| --- | --- | --- | --- |
| All-cause mortality |  |  |  |
| Uric acid, mg/dL | 1.27 (1.24, 1.30) <0.0001 | 1.10 (1.07, 1.13) <0.0001 | 1.06 (1.02, 1.09) 0.0013 |
| Uric acid tertile |  |  |  |
| Low | Reference | Reference | Reference |
| Middle | 1.36 (1.21, 1.53) <0.0001 | 1.00 (0.89, 1.13) 0.9757 | 1.05 (0.93, 1.19) 0.3996 |
| High | 2.02 (1.82, 2.26) <0.0001 | 1.18 (1.05, 1.32) 0.0040 | 1.08 (0.95, 1.23) 0.2648 |
| Cardiovascular mortality |  |  |  |
| Uric acid, mg/dL | 1.36 (1.30, 1.42) <0.0001 | 1.17 (1.12, 1.24) <0.0001 | 1.11 (1.04, 1.17) 0.0011 |
| Uric acid tertile |  |  |  |
| Low | Reference | Reference | Reference |
| Middle | 1.42 (1.14, 1.77) 0.0017 | 1.03 (0.83, 1.29) 0.7687 | 1.04 (0.82, 1.32) 0.7279 |
| High | 2.51 (2.05, 3.08) <0.0001 | 1.42 (1.15, 1.75) 0.0011 | 1.19 (0.93, 1.51) 0.1710 |

Non-adjusted model adjust for: None
Adjust I model adjust for: age; gender; race.
Adjust II model adjust for: age; gender; race; education level; marital status; poverty income ratio; smoking; drinking; BMI; albumin; BUN; eGFR; HBA1c%; FBS; LDL-C; TC; TG; UACR; total calcium; stroke; cancer; hyperlipidemia; hypertension; diabetes; cardiovascular disease; antihypertensive agents; antihyperglycemic agents; CKD Risk; MeTS; CKM syndrome.

Restrict cubic spline smoothing only applies for continuous variables.

Abbreviations: HR, hazard ratio; CI,confidence interval; BMI, body mass index; SBP, systolic blood pressure; DBP, diastolic blood pressure; DM, diabetes mellitus; eGFR, estimated glomerular filtration rate; FBG, fasting blood glucose; HbA1c, hemoglobin A1c; HDL-C, high-density lipoprotein cholesterol; LDL-C, low-density lipoprotein cholesterol; TC, total cholesterol; TG, triglyceride; UACR, urinary albumin creatinine ratio；ALP, alkaline phosphotase; UA, uric acid; BUN, blood urea nitrogen; CKD , Chronic kidney disease；MeTS, metabolic syndrome; CKM, Cardiovascular-Kidney-Metabolic Syndrome.
