# Supplementary material for: Vitamin D as a mediator in the J-shaped association between serum uric acid and all-cause and cardiovascular mortality in patients with cardiovascular–kidney–metabolic syndrome: A prospective cohort study
Source: Medicine (Baltimore). 2026 Jun 26;105(26):e49346. doi: 10.1097/MD.0000000000049346 (PMC13313750; doi:10.1097/MD.0000000000049346)
Supplement: Supplementary file 5 [file medi-105-e49346-s005.docx]

**Table S2 Definition of CKM syndrome staging adjusted for data in NHANES**

| **CKM syndrome stages** | **Definition** |
| --- | --- |
| Stage 0: No CKM health risk factors | Individuals without overweight/obesity, metabolic risk factors (hypertriglyceridemia, hypertension, diabetes, MeTS), CKD or subclinical/clinical CVD  (1)BMI between 18.5 and 25 kg/m^2^, inclusive  (2)WC <102 cm for men or <88 cm for women |
| Stage 1: Excess or dysfunctional adiposity | Individuals with overweight/obesity, abdominal obesity, or adipose tissue dysfunction without other metabolic risk factors, CKD, or subclinical/clinical CVD  (1)BMI ≥ 25 kg/m^2^  (2)WC ≥ 102 cm for men or ≥ 88 cm for women  (3)FBG levels ranging from 100 to 124 mg/dL, or HbA1c levels between 5.7 and 6.4 % |
| Stage 2: Metabolic risk factors and moderate to high-risk CKD | Individuals with metabolic risk factors (hypertriglyceridemia, hypertension, diabetes, MeTS*) or moderate to high-risk CKD stage(The stage of CKD is determined by the KDIGO criteria, using eGFR and UACR(1). The eGFR was calculated using the 2021 race and ethnicity‐free Chronic Kidney Disease Epidemiology Collaboration creatinine equation(2).)  (1)TG >135 mg/dL  (2)Hypertension is defined by an SBP of ≥130 mm Hg, a DBP of ≥80 mm Hg, a medical diagnosis, or taking antihypertensive medication(3).  (3)Diabetes is defined by FBG levels of > 126 mg/dL, HbA1c levels of ≥ 6.5%, a medical diagnosis, or taking insulin or glucose-lowering medication(4).  (4) Moderate to high-risk CKD in the KDIGO classification is defined as UACR ≥ 30 mg/g and eGFR ≥ 60 ml/min/1.73m^2^, UACR < 300 mg/g and eGFR ≤ 45-59 ml/min/1.73m^2^, or UACR < 30 mg/g and eGFR ≤ 30-44 ml/min/1.73m^2^. |
| Stage 3: Subclinical CVD in CKM | Risk equivalents for subclinical CVD: high predicted 10-year CVD risk or very high-risk KDIGO CKD stage  (1)A high 10-year CVD risk is defined as a 20% or above risk, as determined by the basic Predicting Risk of CVD EVENTs (PREVENT) equation(5).  (2)Very high-risk CKD in the KDIGO classification is defined as UACR ≥ 300 mg/g and eGFR ≤ 45-59 ml/min/1.73 m^2^, UACR ≥ 30 mg/g and eGFR ≤ 30-44 ml/min/1.73 m^2^, or eGFR ≤ 29 ml/min/1.73 m^2^. |
| Stage 4: Clinical CVD in CKM | Clinical CVD (self-reported diagnosed cardiovascular disease, including heart failure, coronary heart disease, angina, heart attack, and stroke) in individuals |

Abbreviations: CKM syndrome, Cardiovascular-Kidney-Metabolic syndrome; NHANES, National Health and Nutrition Examination Survey; MeTS, metabolic syndrome; CKD, chronic kidney disease; CVD, cardiovascular disease; BMI, body mass index; WC, waist circumference; FBG, fasting blood glucose; HbA1c, glycated hemoglobin A1c; KDIGO, Kidney Disease Improving Global Outcomes; UACR, urine albumin-to-creatinine ratio; eGFR, estimated glomerular filtration rate; TG, triglycerides; SBP, systolic blood pressure; DBP, diastolic blood pressure.

* MeTS is defined by the presence of ≥3 of the following: (1) WC ≥ 102 cm for men or ≥ 88 cm for women; (2) HDL-C <40 mg/dL for men, <50 mg/dL for women; (3) TG ≥150 mg/dL; (4) Elevated blood pressure (SBP ≥130 mm Hg, DBP ≥80 mm Hg, a medical diagnosis, or taking antihypertensive medication); (5) FBG ≥100 mg/dL

Reference

1. KDIGO 2024 Clinical Practice Guideline for the Evaluation and Management of Chronic Kidney Disease. Kidney international. 2024;105(4s):S117-s314.

2. Inker LA, Eneanya ND, Coresh J, Tighiouart H, Wang D, Sang Y, et al. New Creatinine- and Cystatin C-Based Equations to Estimate GFR without Race. The New England journal of medicine. 2021;385(19):1737-49.

3. Whelton PK, Carey RM. The 2017 American College of Cardiology/American Heart Association Clinical Practice Guideline for High Blood Pressure in Adults. JAMA cardiology. 2018;3(4):352-3.

4. 2. Classification and Diagnosis of Diabetes: Standards of Medical Care in Diabetes-2022. Diabetes care. 2022;45(Suppl 1):S17-s38.

5. Khan SS, Matsushita K, Sang Y, Ballew SH, Grams ME, Surapaneni A, et al. Development and Validation of the American Heart Association's PREVENT Equations. Circulation. 2024;149(6):430-49.
